# Allelopathy of *Lantana camara* as an Invasive Plant

**DOI:** 10.3390/plants10051028

**Published:** 2021-05-20

**Authors:** Hisashi Kato-Noguchi, Denny Kurniadie

**Affiliations:** 1 Department of Applied Biological Science, Faculty of Agriculture, Kagawa University, Miki 761-0795, Kagawa, Japan; 2 Department of Agronomy, Faculty of Agriculture, Universitas Padjadjaran Jl. Raya, Bandung Sumedang KM 21 Sumedang, Jawa Barat 45363, Indonesia; denny.kurniadie@unpad.ac.id

**Keywords:** allelochemical, decomposition, indigenous plant, monospecies stand, phytotoxicity, worst alien species

## Abstract

*Lantana camara* L. (Verbenaceae) is native to tropical America and has been introduced into many other countries as an ornamental and hedge plant. The species has been spreading quickly and has naturalized in more than 60 countries as an invasive noxious weed. It is considered to be one of the world’s 100 worst alien species. *L. camara* often forms dense monospecies stands through the interruption of the regeneration process of indigenous plant species. Allelopathy of *L. camara* has been reported to play a crucial role in its invasiveness. The extracts, essential oil, leachates, residues, and rhizosphere soil of *L. camara* suppressed the germination and growth of other plant species. Several allelochemicals, such as phenolic compounds, sesquiterpenes, triterpenes, and a flavonoid, were identified in the extracts, essential oil, residues, and rhizosphere soil of *L. camara.* The evidence also suggests that some of those allelochemicals in *L. camara* are probably released into the rhizosphere soil under the canopy and neighboring environments during the decomposition process of the residues and as leachates and volatile compounds from living plant parts of *L. camara.* The released allelochemicals may suppress the regeneration process of indigenous plant species by decreasing their germination and seedling growth and increasing their mortality. Therefore, the allelopathic property of *L. camara* may support its invasive potential and formation of dense monospecies stands.

## 1. Introduction

**Figure 1:**
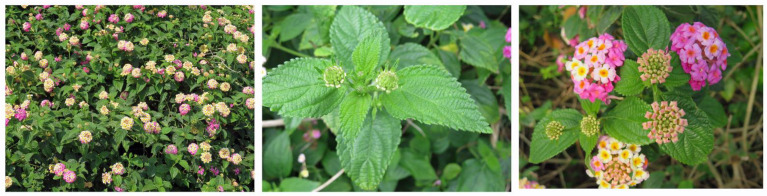
*Lantana camara*. Photos were taken by Kato-Noguchi.

*Lantana camara* L., which belongs to the family of Verbenaceae, is known as wild sage and red sage (Figure 1). It is a perennial shrub 1–4 m tall and forms dense stands. The leaves are opposite with long petioles, oval blades, hairy, and serrate. The species flowers all year round if the condition is adequate. A pair of inflorescences occurs at leaf axils. The flowers are small, multi-colored, and dense in flat-topped clusters. Each inflorescence bears 10–30 fruits, which are small, round drupes containing 1–2 seeds [1,2,3,4].

The species is native to tropical America and has been introduced into other countries as an ornamental and hedge plant. It adapts to varied habitats ranging from open, unshaded areas, such as pastures and crop fields, to disturbed areas, such as roadsides, railway tracks, and fired forests [3,4,5]. The plant has naturalized more than 60 countries as an invasive noxious weed and is considered to be one of the world’s 100 worst invasive alien species [6]. For example, *L. camara* was introduced in India at the beginning of the 19th century and has been growing densely, occupying 13.2 million ha [7]. The plant was first reported in 1841 in Australia and has spread and formed a pure stand over 4 million ha across Australia [8]. The first introduction of the plant in South Africa was in 1858. The species occupied 2 million ha with condensed area of 70,000 ha in 1998 [9]. The species has globally invaded millions of hectares of pastureland and infested major crop plantations, such as tea, coffee, sugarcane, and cotton plantations [3,4]. The invasion of the species also causes the severe reduction of biodiversity in the invaded ecosystems. The species threatened the habitat of 83 indigenous plant species in New South Wales in Australia [10].

*L. camara* displays high morphological variation because of extensive breeding [11]. The genetic diversity of *L. camara* population is high [3,12]. The species has diploid (*n* = 22), triploid (*n* = 33), tetraploid (*n* = 44), and pentaploid (*n* = 55) varieties [13]. Different ploidy levels are of ecological significance in the invasive potential of the species. *L. camara* in the native range of tropical America generally grows as a small clump less than 1 m in diameter. However, it often forms dense monospecies stands in diameter of 1–4 m in the invaded range [2,14]. The requirement conditions for *L. camara* growth and survival are 4.5–8.5 soil pH, 1000–4000 mm annual rainfall, unshaded conditions (however, it is tolerant to shade), and tropical to temperate regions (it is intolerant to frequent freezing) [4]. Thus, the potential of ecological adaptation is very high. Morphological characteristics of the species may contribute to the invasion and naturalization into the nonnative range [15,16,17].

The plants usually flower at the first growing season after establishment in most places [3,18], and there is a range of pollinators, such as insects and birds [19]. It was recorded that one plant produced up to 12,000 fruits each year [20]. Frugivorous birds and other animals contribute to the distribution of the seeds with animal feces, which adds additional nutrients for seed development [19]. *L. camara* also reproduces asexually. Vegetative reproduction occurs by layering horizontal stems and generating root systems [2,21]. The characteristics of *L. camara* for high reproduction may also contribute to the success of its invasion.

The formation of dense monospecies stands of *L. camara,* as described above, indicates its highly competitive ability with other plant species. Perennial plants may be able to release allelochemicals into the soil over several years, and those allelochemicals may accumulate in the soil under the trees [22,23,24,25,26,27]. According to the accumulated literature, *L. camara* possibly releases allelochemicals into the soil under its canopy. Those allelochemicals cause a reduction in seedling recruitment of the native plant species. Subsequently, *L. camara* forms dense monospecies stands. Therefore, allelopathy may play a crucial role in the *L. camara* invasion and formation of monospecies stands. The objective of this review is to discuss the possible involvement of allelopathy in the invasion and establishment of *L. camara.* This review paper provides an overview of the allelopathic property and allelochemicals of *L. camara* and discusses the importance of allelopathy for the invasion and formation of dense monospecies stands of *L. camara.*

## 2. Allelopathy of *L. camara*

Allelopathy is the interaction of one plant with another plant in its vicinity through releasing certain secondary metabolites, which are defined as allelochemicals [28]. The allelochemicals are released into the neighboring environments and rhizosphere soil of the plants by rainfall leachates, decomposition of plant residues, root exudation, and volatilization from living plant parts [28,29,30,31]. Therefore, the allelopathic potential of the extracts, leachates, residues, and rhizosphere soil of *L. camara* was evaluated by many researchers. In this chapter, the allelopathic potential of the extracts, leachates, residues, and rhizosphere soil of *L. camara* is summarized (Table 1).

### 2.1. Extract

Aqueous leaf extracts of *L. camara* inhibited the germination of *Lactuca sativa* L. due to the suppression of cellular membrane developments and increase in the production of reactive oxygen forms [32]. Aqueous leaf extracts of *L. camara* suppressed the development of leaf buds of *Eichhornia crassipes* Mart., increased the decay, and caused necrosis of *E. crassipes* leaves. The extracts also increased SOD activity in the leaves of *E. crassipes* concomitant with H_2_O_2_ accumulation and increased the membrane peroxidation level. The activity of catalase was decreased by the extract treatments. These observations indicate that leaf necrosis of *E. crassipes* may occur due to the oxidative stress caused by leaf extracts of *L. camara* [33].

Aqueous leaf extracts of *L. camara* inhibited the germination and growth of *Brassica juncea* (L.) Czern, *Cucumis sativus* L., *Phaseolus mungo* L., *Raphanus sativus* L., *Vigna unguiculata* (L.) Walp, *Cicer arietinum* L. [34], *Centroma pubescens* Benth. [35], and *Vigna radiata* (L.) R. Wilczek [36]. Aqueous extracts of *L. camara* leaves, stems, and roots also suppressed the germination and growth of *Cicer arietinum* L. [37], *Phaseolus mungo* (L.) Hepper [38], and *Lens esculenta* Moench [39]. In addition, the aqueous leaf extracts suppressed the regeneration of the moss species *Funaria hygrometrica* Hedw. [40].

Aqueous extracts of *L. camara* flowers suppressed the germination and seedling growth of *Eruca sativa* (L.) Cav. [41]. Aqueous extracts of flowers, fruits, and leaves inhibited the germination and seedling growth of *Raphanus sativus* L. and *Lactuca sativa* L. The inhibitory effect was more significant with flower and fruit extracts than leaf extracts [42]. Methanol extracts of stem and leaves of *L. camara* also inhibited the germination and growth of *Lolium multiflorum* Lam. [43]. The observations described in this section indicate that the extracts of *L. camara* possess inhibitory activity on the germination and growth of several other plant species and probably contain some extractable allelochemicals.

### 2.2. Leachate

Shoots and flowers of *L. camara* were cut into small pieces and soaked in water for 48 h. Filtered water was used as the leachates of *L. camara*. The leachates inhibited the growth of *Eichhornia crassipes* Mart. and finally killed *E. crassipes* 21 days after the treatment due to its high toxicity [44,45]. Leachates from *L. camara* leaves suppressed the germination and seedling growth of *Mimosa pudica* L. The concentrations of insoluble carbohydrate, proteins, and nucleic acids and the activities of dehydrogenase, catalase, and peroxidase in the seedlings were reduced by the leachates. However, the concentrations of amino acids and soluble carbohydrates were increased by the treatment [46,47]. Root leachates of *L. camara* inhibited the radical growth of *Cucurbita pepo* Linnaeus, *Phaseolus vulgaris* L., and *Lycopersicon esculentum* Mill. and altered the cytoplasmic protein synthesis in those radicals [48]. Leachates from *L. camara* roots also suppressed the germination and seedling vigor of *Triticum aestivum* L. [49]. Leachates from fruits and leaves of *L. camara* significantly inhibited the growth of *Pennisetum americanum* (L.) Tzvelev, *Setaria italica* (L.) P. Beauvois, and *Lactuca sativa* L. [50]. These observations suggest that some allelochemicals may be released into the soil under the trees from the leaves, shoots, flowers, fruits, and roots of *L. camara* by rain and irrigation water as leachates.

### 2.3. Residue

*L. camara* shoots were cut into small pieces and mixed with sand, and the seeds of *Triticum aestivum* L., *Zea mays* L., *Glycine max* (L.) Merr., *Lepidium virginicum* L., and *Abutilon theophrasti* Medik. were sown into the mixture. The growth of those seedlings was significantly suppressed by the residues of *L. camara* [51]. Lantana root and shoot residues and those decomposed residues also suppressed the growth of *Morrenia odorata* (Hook. & Arm.) Lindi. [52]. Decomposed leaf litter of *L. camara* inhibited the seedling growth of *Raphanus sativus* L., *Lactuca sativa,* L. *Bidens pilosa* L., *Bidens bipinnata* L., and *Urena lobata* L. [53]. These findings indicate that some allelochemicals were released into the soil during the decomposition process of *L. camara* residues.

### 2.4. Rhizosphere Soil

Rhizosphere soil of *L. camara* suppressed the growth of *Achyranthes aspera* L. and *Albizia lebbeck* [54]. Rhizosphere soil of *L. camara* also reduced the germination and seedling growth of *Avena sativa* L., *Cicer arietinum* L., *Hordeum vulgare* L., and *Triticum aestivum* L. [55]. These observations suggest that the rhizosphere soil of *L. camara* may contain some allelochemicals. The allelochemicals may occur during the decomposition process of plant residues in the soil and/or as leachates from living plant parts and root exudation.

**Table 1:** Allelopathic activities of the extracts, leachates, residues, and rhizosphere soil of *Lantana camara*.

Source	Target Plant Species	Inhibition	Stimulation	Reference
Extract				
Leaf	*Lactuca sativa*	Germination, cellular membrane development	Reactive oxygen form	[32]
	*Eichhornia crassipes*	Development of leaf buds, catalase, leaf necrosis	SOD activity, H_2_O_2_ accumulation, membrane peroxidation	[33]
	*Brassica juncea, Cucumis sativus, Phaseolus mungo, Raphanus sativus, Vigna unguiculata, Cicer arietinum*	Germination and growth		[34]
	*Centroma pubescens*	Germination and growth		[35]
	*Vigna radiata*	Germination and growth		[36]
	*Funaria hygrometrica*	Regeneration		[40]
Leaf, stem	*Lolium multiflorum*	Germination and growth		[43]
Leaf, stem, root	*Cicer arietinum,*	Germination and growth		[37]
	*Phaseolus mungo*	Germination and growth		[38]
	*Lens esculenta*	Germination and growth		[39]
Flower	*Eruca sativa*	Germination and growth		[41]
Flower, fruit, leaf	*Raphanus sativus, Lactuca sativa*	Germination and growth		[42]
Leachate				
Shoot, flower	*Eichhornia crassipes*	Growth		[44,45]
Leaf	*Mimosa pudica*	Concentrations of insoluble carbohydrate, protein and nucleic acid. Activities of dehydrogenase, catalase and peroxidase	Concentrations of amino acid and soluble carbohydrate	[46,47]
Root		Growth, protein synthesis		[48]
	*Triticum aestivum,*	Germination and growth		[49]
Fruit, leaf	*Pennisetum americanum, Setaria italica, Lactuca sativa*	Growth		[50]
Residue				
Shoot	*Triticum aestivum, Zea mays, Glycine max, Lepidium virginicum, Abutilon theophrasti*	Growth		[51]
Root, shoot, decomposed	*Morrenia odorata*	Growth		[52]
Decomposed leaf litter	*Raphanus sativus, Lactuca sativa, Bidens pilosa, Bidens bipinnata, Urena lobata*	Growth		[53]
Rhizosphere soil	*Achyranthes aspera, Albizia lebbeck*	Growth		[54]
	*Avena sativa, Cicer arietinum., Hordeum vulgare, Triticum aestivum*	Germination and growth		[55]

## 3. Allelochemicals

Aqueous extracts of *L. camara* leaves inhibited the germination and seedling growth of *Lolium multiflorum* Lam., and 13 phenolic compounds were identified in the extracts. Those compounds were caffeic acid, gentisic acid, *p*-hydroxybenzoic acid, vanillic acid, salicylic acid, ferulic acid, *p*-coumaric acid, methyl coumarin, α-resorcylic acid, β-resorcylic acid, vanillin, and quercetin. All compounds inhibited the growth of *L. multiflorum.* The inhibitory activity of salicylic acid and methyl coumarin was more significant than that of other compounds [56]. Aqueous extracts of *L. camara* leaves also inhibited the growth of *Lemna minor* L., and 14 phenolic compounds: caffeic acid, gentisic acid, *p*-hydroxybenzoic acid, vanillic acid, salicylic acid, ferulic acid, *p*-coumaric acid, *m*-coumaric acid, *o*-coumaric acid, methyl coumarin, syringic acid, protocatechuic acid, *t*-cinnamic acid, and vanillin were identified in the extracts. The main phenolic compound in the extracts was *p*-coumaric acid, and the most active compound in the extract was salicylic acid [57].

Lantadene A and lantadene B were isolated from aqueous methanol extracts of *L. camara* leaves as allelochemicals [58]. Both compounds were first isolated as toxic substances in *L. camara* [59]. Lantadene A and lantadene B inhibited *Eichhornia a* Mart. and *Microcystis aeruginosa* (Kützing) Kützing at concentrations greater than 13.7 mg L^−1^ and 10.8 mg L^−1^, respectively. Lantadene A and lantadene B were also found in rhizosphere soil of *L. camara* at phytotoxic active levels [58]. The concentrations of those compounds varied by the geographical origin of the plants between trace amounts to 2.2% of leaves’ dry weight [60,61]. Methanol extracts of *L. camara* leaves also inhibited the growth of *Phalaris minor* Retz, *Avena fatua* L., *Chenopodium album* L., and *Rumex dentatus* L., and the flavone glucoside vitexin was isolated from the extracts as an allelochemical of *L. camara* [62].

The essential oil obtained from *L. camara* leaves suppressed the growth of plant pathogenic fungi *Corynespora cassiicola* (Berk. & M.A.Curtis) C.T. Wei. The main volatile compounds in the essential oil were germacrene D (19.8% of total oil), *E*-caryophyllene (19.7%), bicyclogermacrene (11.7%), and α-humulene (9.3%) [63]. The essential oil obtained from *L. camara* leaves also suppressed the seedling growth of *Portulaca oleracea* L. The major volatile compounds of the oil were β-caryophyllene, α-humulene, γ-muurolene, α-curcumene, β-curcumene, and γ-curcumene [64]. Some of the volatile compounds, such as β-caryophyllene, α-humiulene, and *E*-caryophyllene, have growth inhibitory activity [63,64]. Allelochemicals found in *L. camara* are summarized in Table 2.

A number of secondary metabolites in many classes, such as monoterpenes, sesquiterpenes, diterpenes, triterpenes, iridoid glycosides, flavonoids, and phenylethanoid glycosides, have been isolated and identified in the fruits, flowers, leaves, stems and roots of *L. camara* [61,64,65,66]. The biological activity of some of those compounds has been associated with anti-microbial, anti-fungal, anti-herbivore, and pharmacological activities. The allelopathic activity of most of the identified compounds in *L. camara* has not yet been determined. However, some of those compounds may possess phytotoxic activity [61,64,65,66]. Phytotoxic substances in plants can be released into the rhizosphere soil under the plants and neighboring environments either by rainfall leachates and volatile compounds from living plant parts of *L. camara* or by decomposition of plant residues and act as allelochemicals [29,30,31]. As described the previous section, the leachates from the plant parts of *L. camara*, residues, and rhizosphere soil *L. camara* suppressed the germination and growth of several other plant species. Therefore, allelochemicals of *L. camara* are probably released into its rhizosphere soil under the canopy and neighboring environments.

## 4. Invasion and Allelopathy of *L. camara*

The invasion of *L. camara* significantly reduced the plant diversity in the invaded area. The biomass of the *L. camara* invaded area was about half of that of the uninvaded areas [54]. *Albizia lebbeck* (L.) Benth. Is an indigenous plant species in Siwalik Hills in northern India, but no seedling was observed in the *L. camara* invaded areas. *L. camara* suppressed the growth and seedling recruitment of almost all native plant species under its canopy [3,67]. Rhizosphere soil of *L. camara* also significantly suppressed the growth of native plant species *Achyranthes aspera* L. [54]. In addition, activated charcoal recovered the inhibitory effect of the rhizosphere soil of *L. camara* [3]. These observations suggest that the rhizosphere soil of *L. camara* may contain some allelochemicals that disturb the regeneration process of native plant species by decreasing their germination and seedling growth and increasing their mortality.

**Figure 2:**
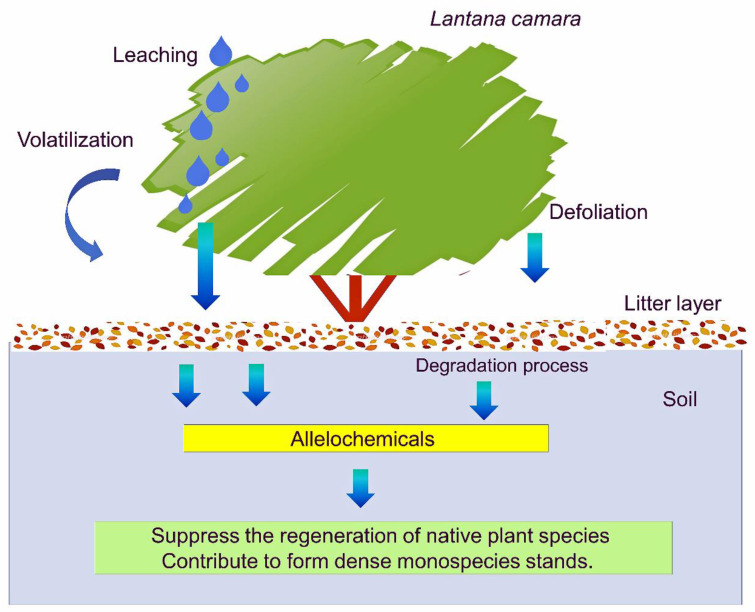
Allelopathy of *Lantana camara* contributes to forming dense monospecies stands.

*L. camara* is a perennial shrub, but senescent leaves are replaced by new leaves [3,4]. Those dropped leaves accumulate on the soil under the canopy and form a litter layer. Some of the secondary metabolites in the leaves may be liberated into rhizosphere soil during the decomposition process of the litter and act as allelochemicals (Figure 2). Allelochemicals are able to inhibit germination, seedling establishment, and plant growth of other plant species [29,30,31]. A substantial number of allelochemicals were identified in the *L. camara* leaves (Table 2). In addition, the decomposition rate of *L. camara* litter was faster than that of the leaf litter of native plant species [68]. In fact, lantadene A and lantadene B, which were isolated from the leaves, were also found in the rhizosphere soil of *L. camara* over growth-inhibitory levels [58]. The concentration of total phenolic compounds in the rhizosphere soil of *L. camara* was 27.6% higher than that in the soil of the uninvaded area of *L. camara* [69]. Phenolic compounds are often mentioned as putative allelochemicals, as they have been found in a wide range of plants and soils [70,71]. The inhibitory activity of most phenolic compounds is concentration dependent. Phenolic compounds interfere with several enzyme activities and almost all of the major physiological processes, such as phytohormone functions, mineral uptake, water balance and stomatal functions, photosynthesis, respiration, and the metabolism of certain compounds and flow carbons [70,72]. Those observations indicate that allelochemicals in *L. camara* litter may be released into rhizosphere soil during the decomposition process.

Many of the phytotoxic substances from the invasive plants have been reported to have multiple functions, such as anti-pathogen, anti-herbivore, and allelopathic activity [17,73,74]. Those compounds probably provide the plants with the advantage of increasing their population in the new habitats. A large number of secondary metabolites have been isolated from the extracts of different parts of *L. camara*, including monoterpenes, sesquiterpenes, diterpenes, triterpenes, iridoid glycosides, flavonoids, and phenylethanoid glycosides, and some of them were reported to possess anti-microbial, anti-insect, and pharmacological activities. [61,64,65,66]. Therefore, some of these compounds probably enhance the competitive ability of *L. camara* and make the plant invasive.

The characteristics of life history, such as high reproduction and high growth rate of *L. camara*, are essential to the success of the invasion into nonnative ranges [15,16,17]. The genetic diversity of *L. camara* is crucial for phenotypic plasticity to adapt to different environmental conditions [3,12,13]. Competitive ability is also one of the essential factors for *L. camara* to naturalize and establish dense monospecies stands in nonnative ranges [1,2,3,4]. As described previously, *L. camara* interrupts the regeneration process of indigenous plant species by decreasing their germination, reducing their seedling growth, and increasing their mortality. *L. camara* is allelopathic, and its allelopathic property may help the invasion of this species into nonnative ranges. In addition, the elevated temperature increased the allelopathy of *L. camara* [75], which indicates that global warming may increase the threat of the invasion of the species into additional nonnative areas. 

## 5. Conclusions

*L. camara* is very invasive and often forms dense monospecies stands through the interruption of the regeneration process of native plant species by decreasing their germination and seedling growth and increasing their mortality. The evidence summarized in this paper indicates that *L. camara* is allelopathic (Table 1) and contains several allelochemicals, such as phenolic compounds, sesquiterpenes, triterpenes, and a flavonoid (Table 2). The evidence also suggests that some of those allelochemicals in *L. camara* are probably released into the rhizosphere soil under its canopy and neighboring environments during the decomposition process of its residues and as leachates and volatile compounds from living plant parts of *L. camara* (Figure 2). Allelochemicals can inhibit the germination and growth of other plant species. Therefore, the allelochemicals released from *L. camara* may provide the species with a competitive advantage against the native plants and may also suppress the regeneration process of native plant species and contribute to establishing their habitats as invasive plant species. However, it is essential to specify the allelochemical and to clarify its release process and quantity into the neighboring environments to understand the invasion and formation of dense monospecies stands of *L. camara*.

## Figures and Tables

**Table 2:** Allelochemicals of *Lantana camara* and target plant species for their activity.

Allelochemical	Chemical Class	Source	Target Plant Species	Inhibition	Reference
Caffeic acid, gentisic acid, *p*-hydroxybenzolic acid, vanillic acid, salicylic acid, ferulic acid, *p*-coumaric acid, methyl coumarin, α-resorcylic acid, β-resorcylic acid, vanillin, quercetin.	Phenolic	Leaf	*Lolium multiflorum*	Growth	[56]
Caffeic acid, gentisic acid, *p*-hydoxybenzoic acid, vanilic acid, salicylic acid, ferulic acid, *p*-coumaric acid, *m*-coumaric acid, *o*-coumaric acid, methyl coumarin, syringic acid, protocatechuic acid, *t*-cinnamic acid and vanillin	Phenolic	Leaf	*Lemna minor*	Growth	[57]
Lantadene A, lantadene B	Triterpene	Leaf, rhizosphere soil	*Eichhornia crassipeas, Microcystis aeruginosa*	Growth	[58]
Vitexin	Flavonoide	Leaf	*Phalaris minor, Avena fatua, Chenopodium album, Rumex dentatus*	Growth *	[62]
*E*-caryophyllene, bicyclogermacrene, α-humulene	Sesquterpene	Essential oil from leaf	*Corynespora cassiicola*	Growth **	[63]
β-Caryophyllene, α-humulene, γ-muurolene, α-curcumene, β-curcumene, γ-curcumene	Sesquterpene	Essential oil from leaf	*Portulaca oleracea*	Growth **	[64]

Inhibitory activity was determined with a crude extract * and with total essential oil **.

## References

[1] Duggin J.A., Gentle C.B. (1998). Experimental evidence on the importance of disturbance intensity for invasion of *Lantana camara* L. in dry rainforest-open forest ecotones in north-eastern NSW, Australia. For. Ecol. Manag..

[2] Swarbrick J.T., Willson B.W., Hanna-Jones M.A., Panetta F.D., Groves R.H., Shepherd R.C.H. (1998). *Lantana camara* L.. The Biology of Australian Weeds.

[3] Sharma G.P., Raghubanshi A.S., Singh J.S. (2005). Lantana invasion: An overview. Weed Biol. Manag..

[4] Priyanka N., Joshi P.K. (2013). A review of *Lantana camara* studies in India. Int. J. Sci. Res. Pub..

[5] Dogra K.S., Kohli R.K., Sood S.K. (2009). An assessment and impact of three invasive species in the Shivalik hills of Himachal Pradesh, India. Int. J. Biodivers. Conserv..

[6] Global Invasive Species Database Species Profile: *Lantana camara*. http://www.iucngisd.org/gisd/species.php?sc=56.

[7] Singh S.P., Paroda R.S., Chadha K.L. (1996). Biological control. 50 Years of Crop. Science Research in India.

[8] Van Oosterhout E., Clark A., Day M.D., Menzies E. Lantana Control Manual. Current Management and Control. Options for Lantana (*Lantana camara*) in Australian State of Queensland. http://www.nrm.qld.gov.au/pests/wons/Lantana.

[9] Vardien W., Richardson M.D., Foxcroft L.C., Thompson G.D., Wilson J.R.U., Le Rouxa J.J. (2012). Invasion dynamics of *Lantana camara* L. (sensu lato) in South Africa. S. Afr. J. Bot..

[10] Coutts-Smith A., Downey P. (2006). Impact of Weeds on Threatened Biodiversity in New South Wales.

[11] Binggeli P., Goodman S.M., Benstead J.P. (2003). Verbenaceae, *Lantana camara*, fankatavinakoho, fotatra, mandadrieko, rajejeka, radredreka, ramity. The Natural History of Madagascar.

[12] Peng Z., Bhattarai K., Parajuli S., Cao Z., Deng Z. (2019). Transcriptome analysis of young ovaries reveals candidate genes involved in gamete formation in *Lantana camara*. Plants.

[13] Everist S.L. (1981). Poisonous Plants of Australia.

[14] Palmer W.A., Pullen K.R. (1995). The phytophagous arthropods associated with *Lantana camara*, *L. hirsuta*, *L. urticifolia* and *L. urticoides* (Verbenaceae) in North America. Biol. Control.

[15] Thompson J.D., McNeilly T., Gray A.J. (1991). Population variation in *Spartina anglica* C.E. Hubbard: I. Evidence from a common garden experiment. New Phytol..

[16] Mack R.M. (1996). Predicting the identity and fate of plant invaders: Emergent and emerging approaches. Biol. Conserv..

[17] Cappuccino N., Arnason J.T. (2006). Novel chemistry of invasive exotic plants. Biol. Lett..

[18] Gujral G.S., Vasudevan P. (1983). *Lantana camara* L., a problem weed. J. Sci. Indust. Res..

[19] Jordaan L.A., Johnson S.D., Downs C.T. (2011). The role of avian frugivores in germination of seeds of fleshy-fruited invasive alien plants. Biol. Inva..

[20] Mack R.N., Simberloff D., Lonsdale W.M., Evans H., Clout M., Bazzaz F.A. (2000). Biotic invasions: Causes, epidemology, global consequences and control. Ecol. Appl..

[21] Swarbrick J.T., Willson B.W., Hannan-Jones M.A. (1995). The biology of Australian weeds 25 *Lantana camara* L.. Plant Prot. Q..

[22] Mallik A.U. (1998). Allelopathy and competition in coniferous forests. Environ. For. Sci..

[23] Kato-Noguchi H., Takeshita S., Kimura F., Ohno O., Suenaga K. (2013). A novel allelopathic active substance in *Ginkgo biloba*. J. Plant Physiol..

[24] Kato-Noguchi H., Takeshita S. (2013). Contribution of a phytotoxic compound to the allelopathy of *Ginkgo biloba*. Plant Signal. Behav..

[25] Kato-Noguchi H., Kimura F., Ohno O., Suenaga K. (2017). Involvement of allelopathy in inhibition of understory growth in red pine forests. J. Plant Physiol..

[26] Kato-Noguchi H., Kurniadie D. (2020). Allelopathy and allelopathic substances of mango (*Mangifera indica* L.). Weed Biol. Manag..

[27] Kato-Noguchi H. (2021). Phytotoxic substances involved in teak allelopathy and agroforestry. Appl. Sci..

[28] Rice E.L. (1984). Allelopathy.

[29] Bais H.P., Weir T.L., Perry L.G., Gilroy S., Vivanco J.M. (2006). The role of root exudates in rhizosphere interactions with plants and other organisms. Annu. Rev. Plant Biol..

[30] Bonanomi G., Sicurezza M.G., Caporaso S., Esposito A., Mazzoleni S. (2006). Phytotoxicity dynamics of decaying plant materials. New Phytol..

[31] Belz R.G. (2007). Allelopathy in crop/weed interactions—An update. Pest Manag. Sci..

[32] Gindri D.M., Coelho C.M.M., Uarrota V.G. (2020). Physiological and biochemical effects of *Lantana camara* L. allelochemicals on the seed germination of *Avena sativa* L.. Pesqui. Agropecu. Trop..

[33] Zheng H., Wei N., Wang L., He P. (2006). Effects of *Lantana camara* leaf extract on the activity of superoxide dismutase and accumulation of H_2_O_2_ in water hyacinth leaf. J. Plant Physiol. Mol. Biol..

[34] Ahmed R., Uddin M.B., Khan M.A., Mukul S.A., Hossain M.K. (2007). Allelopathic effects of *Lantana camara* on germination and growth behavior of some agricultural crops in Bangladesh. J. For. Res..

[35] Rusdy M., Ako A. (2017). Allelopathic effect of *Lantana camara* and *Chromolaena odorata* on germination and seedling growth of *Centroma pubescens*. Int. J. Appl. Environ. Sci..

[36] Julio A., Tandoc W.C., Tipace H.D., Vendivil Y.F., Yanesa Z., Tare M.V.R., Lactaoen E.J., Clemente K.J. (2019). Allelopathic effect of *Lantana camara* and *Chromolaena odorata* leaf extracts on plant germination. Asian J. Agric. Biol..

[37] Oudhia P. (2000). Allelopathic effects of *Lantana camara* L. on chickpea. Ecol. Environ. Conserv..

[38] Vijay B., Jain B.K. (2010). Allelopathic effects of *Lantana camara* L. on in vitro seed germination of *Phaseolus mungo*. Int. J. Plant Sci..

[39] Singh A., Satsangi G.P., Srivastava J.N. (2012). Allelopathic aspects of *Lantana camara* on germination and seedling growth of *Lens esculanta*. Vegetos.

[40] Choyal R., Sharma S.K. (2011). Allelopathic effects of *Lantana camara* (Linn) on Regeneration in *Funaria hygrometrica*. Indian J. Fund. Appl. Life Sci..

[41] Labruzzo A., Carrubba A., Di Marco G., Ebadi M.T. (2017). Herbicidal potential of aqueous extracts from *Melia azedarach* L., *Artemisia arborescens* L., *Rhus coriaria* L. and *Lantana camara* L.. Allelopath. J..

[42] Zhang Q., Peng S., Zhang Y. (2009). Allelopathie potential of reproductive organs of exotic weed *Lantana camara*. Allelopath. J..

[43] Achhireddy N.R., Singh M., Achhireddy L.L., Nigg H.N., Nagy S. (1985). Isolation and partial characterization of phytotoxic compounds from lantana (*Lantana camara* L.). J. Chem. Ecol..

[44] Saxena M.K. (2000). Aqueous leachate of *Lantana camara* kills water hyacinth. J. Chem. Ecol..

[45] Motwani G., Golani N., Solanki H. (2013). Allelopathic effects of aqueous leaf leachates of *Lantana camara* on *Eichhorina crassipes*. Lifesci. Leafl..

[46] Maiti P.P., Bhakat R.K., Bhattacharjee A. (2008). Allelopathic effects of *Lantana camara* on physio-biochemical parameters of *Mimosa pudica* seeds. Allelopath. J..

[47] Maiti P. (2020). Biometric evaluation of allelopathic potential of *Lantana camara* L. on *Mimosa* seeds. J. Crit. Rev..

[48] Romero-Romero T., Anaya A.L., Cruz-Ortega R. (2002). Screening for effects of phytochemical variability on cytoplasmic protein synthesis pattern of crop plants. J. Chem. Ecol..

[49] Oudhia P. (2001). Allelopathic effects of root leachates of some obnoxious weeds on germination and seedling vigour of wheat. Ecol. Environ. Conserv..

[50] Hussain F., Ghulam S., Sher Z., Ahmad B. (2011). Allelopathy by *Lantana camara* L.. Pak. J. Bot..

[51] Mersie W., Singh M. (1987). Allelopathic effect of lantana on some agronomic crops and weeds. Plant Soil.

[52] Achhireddy N.R., Singh M. (1984). Allelopathic effects of lantana (*Lantana camara*) on milkweedvine (*Morrenia odorata*). Weed Sci..

[53] Wang R., Kang X., Quan G., Zhang J. (2015). Influence of *Lantana camara* on soil II. Effects of *Lantana camara* leaf litter on plants and soil properties. Allelopath. J..

[54] Singh H.P., Batish D.R., Dogra K.S., Kaur S., Kohli R.K., Negi A. (2014). Negative effect of litter of invasive weed *Lantana camara* on structure and composition of vegetation in the lower Siwalik Hills, northern India. Environ. Monit. Assess..

[55] Hayyat M.S., Safdar M.E., Asif M., Tanveer A., Ali L., Qamar R., Ali H.H., Farooq N., Javeed H.M.A., Tarar Z.H. (2020). Allelopathic effect of waste-land weeds on germination and growth of winter crops. Planta Daninha.

[56] Singh M., Tamma R.V., Nigg H.N. (1989). HPLC identification of allelopathic compounds from *Lantana camara*. J. Chem. Ecol..

[57] Jain R., Singh M., Dezman D.J. (1989). Qualitative and quantitative characterization of phenolic compounds from lantana (*Lantana camara*) leaves. Weed Sci..

[58] Kong C.H., Wang P., Zhang C.X., Zhang M.X., Hu F. (2006). Herbicidal potential of allelochemicals from *Lantana camara* against *Eichhornia crassipes* and the alga *Microcystis aeruginosa*. Weed Res..

[59] Barton D.H.R., de Mayo P. (1954). Triterpenoids. Part XVI. Theconstitution of rehmannic acid. J. Chem. Soc..

[60] Hart N., Lamberton J., Sioumis A., Suares H. (1976). New triterpenes of *Lantana camara*. A comparative study of the constituents of several taxa. Aust. J. Chem..

[61] Ghisalberti E.L. (2000). *Lantana camara* L. (Verbenaceae). Fitoterapia.

[62] Qureshi H., Anwar T., Ali Q., Haider M.Z., Habib N., Fatima S., Waseem M., Bibi Y., Arshad M., Adkins S.W. (2021). Isolation of natural herbicidal compound from *Lantana camara*. Int. J. Environ. Anal. Chem..

[63] Passos J.L., Barbosa L.C.A., Demuner J.A., Alvarenga E.S., da Silva C.M., Barreto R.W. (2012). Chemical characterization of volatile compounds of *Lantana camara* L. and *L. radula* Sw. and their antifungal activity. Molecules.

[64] Verdeguer M., Blázquez M.A., Boira H. (2009). Phytotoxic effects of *Lantana camara*, *Eucalyptus camaldulensis* and *Eriocephalus africanus* essential oils in weeds of Mediterranean summer crops. Biochem. Syst. Ecol..

[65] Sharma O.P., Sharma S., Pattabhi V., Mahato S.B., Sharma P.D. (2007). A review of the hepatotoxic plant *Lantana camara*. Crit. Rev. Toxicol..

[66] Mishra A. (2015). Allelopathic properties of *Lantana camara*. Int. Res. J. Basic Clin. Stud..

[67] Gentle C.B., Duggin J.A. (1997). Allelopathy as a competitive strategy in persistent thickets of *Lantana camara* L. in three Australian forest communities. Plant Ecol..

[68] Mutshekwa T., Cuthbert R.N., Wasserman R.J., Murungweni F.M., Dalu T. (2020). Macroinvertebrate colonisation associated with native and invasive leaf litter decomposition. Knowl. Manag. Aquat. Ecosyst..

[69] Seastedt T.R., Callaway R.M., Pollock J.L., Kaur J. (2008). Allelopathy and plant invasions: Traditional, congeneric, and bio-geographical approaches. Biol. Inva..

[70] Inderjit (1996). Plant phenolics in allelopathy. Bot. Rev..

[71] Dalton B.R., Inderjit, Dakshini K.M.M., Foy C.L. (1999). The occurrence and behavior of plant phenolic acids in soil environments and their potential involvement in allelochemical interference interactions: Methodological limitations in establishing conclusive proof of allelopathy. Principals and Practices in Plant Ecology: Allelochemical Interactions.

[72] Einhellig F.A., Macías F.A., Galindo J.C.G., Molino J.M.G., Cutler H.G. (2004). Mode of action of allelochemical action of phenolic compounds. Chemistry and Mode of Action of Allelochemicals.

[73] Lockwood J.L., Simberloff D., McKinney M.L., Von Holle B. (2001). How many, and which, plants will invade natural areas. Biol. Inva..

[74] Wang C., Zhu M., Chen X., Bo Q. (2011). Review on allelopathy of exotic invasive plants. Procedia Eng..

[75] Zhang Q., Zhang Y., Peng S., Zobel K. (2014). Climate warming may facilitate invasion of the exotic shrub *Lantana camara*. PLoS ONE.

